# Reporting a rare form of myopathy, myopathy with extrapyramidal signs, in an Iranian family using next generation sequencing: a case report

**DOI:** 10.1186/s12881-020-01016-y

**Published:** 2020-04-15

**Authors:** Marzieh Mojbafan, Somayeh Takrim Nojehdeh, Faezeh Rahiminejad, Yalda Nilipour, Seyed Hasan Tonekaboni, Sirous Zeinali

**Affiliations:** 1 Department of Medical Genetics and Molecular Biology, Faculty of Medicine, Iran University of Medical Sciences (IUMS), Shahid Hemmat Highway, Tehran, Iran; 2 Department of medical genetics, Ali-Asghar Children’s Hospital, Zafar St., Shahid Modarres Highway, Tehran, Iran; 3 Medical Genetics Lab, Kawsar Human Genetics Research Center, No. 41, Majlesi St., Valieasr Ave, Tehran, Iran; 4 Pathology Department, Pediatric Pathology Research Center, Research Institute for Children Health, Mofid Hospital, Shahid Beheshti University of Medical Sciences, Tehran, Iran; 5 Pediatric Neurology Center of Excellence, Department of Pediatric Neurology, Mofid Children Hospital, Faculty of Medicine, Shahid Beheshti Medical University, Tehran, Iran; 6 Department of Molecular Medicine, Biotechnology Research Center, Pasteur Institute of Iran, No. 69, Pasteur Ave, Tehran, Iran

**Keywords:** Myopathy with extrapyramidal signs, *MICU1* gene, Next generation sequencing, Autozygosity mapping

## Abstract

**Background:**

Myopathy with extrapyramidal signs (MPXPS) is an autosomal recessive mitochondrial disorder which is caused by mutation in mitochondrial calcium uptake 1 (*MICU1)* gene located on chromosome 10q22.1. Next Generation Sequencing (NGS) technology is the most effective method for identification of pathogenic variants with the ability to overcome some limitations which Sanger sequencing may encountered. There are few reports on this rare disease around the world and here in this study we first revealed genetic identification of two affected individuals in an Iranian family with a novel mutation.

**Case presentation:**

The proband was a 5-year-old girl from consanguenous parents. She was first clinically suspicious of affected with limb-girdle muscular dystrophy (LGMD). Muscle biopsy studies and autozygosity mapping, using four short tandem repeat (STR) markers linked to 6 genes of the most prevalent forms of LGMD, ruled out calpainopathy, dysferlinopathy, and sarcoglycanopathis. DNA sample of the proband was sent for NGS. Whole exome sequencing (WES**)** revealed a novel mutation c.1295delA in exon 13 of *MICU1* gene. This homozygous deletion creates a frameshift and a premature stop codon downstream of canonical EF4 calcium binding motif of MICU1. According to the American College of Medical Genetics and Genomics (ACMG) guidline for sequence interpretation, this variant was a pathogenic one. Sanger sequencing in all family members confirmed the results of the WES.

**Conclusions:**

This study was the first report of MPXPS in Iranian population which also revealed a novel mutation in the *MICU1* gene**.**

## Background

Myopathy with extrapyramidal signs (OMIM #615673) is a rare autosomal recessive mitochondrial disorder caused by mutations in *MICU1* gene that typically causes muscle weakness with normal respiratory chain [1, 2]. *MICU1* (OMIM #605084) which is also known as *CALC, EFHA3, MPXPS* and *CBARA1* gene, is located on chromosome 10q22.1 and includes 17 exons [3–5]. This gene is translated into a 476 amino acid protein with ~ 54 kDa molecular mass [6]. MICU1 protein is an inner mitochondrial membrane (IMM) protein with three distinct regions: N-terminal domains (residues 1–32) consisting of mitochondrial targeting sequence and MICU1/MCU complex binding polybasic domain, a hydrophobic transmembrane α-helix domain (TM) (residues 33–52), and cytosolic C-terminal Ca^2+^ binding domain (residues 53–476) [6]. The X-ray crystallography of the Ca^2+^-free MICU1 revealed that C-terminal domain is divided into four constructs including the N-domain (aa ~ 103–177), the N-lobe (aa ~ 183–318), the C-lobe (aa ~ 319–445), and the C-helix (aa ~ 446–476) that canonical EF1 and EF4 hands are responsible for binding to cytosolic Ca^2+^ which are located in the N-lobe and C-lobe, respectively [6, 7].

Few studies showed pathogenic variants in the *MICU1* gene in patients from European and middle East countries [1, 8, 9].

LGMD is a large heterogeneous group of neuromuscular disorders which can be transmitted in autosomal dominant (LGMDD) and autosomal recessive (LGMDR) manner [10, 11]. Common feature of LGMD disorders is the progressive weakness of the shoulder girdle muscles [11].

Autozygosity mapping is a good and practical approach in the gene tracking of heterogeneous disorders in consanguineous families [12, 13]. Since the cost of next generation sequencing continues to decreasethe NGS will be widly used in identifying pathogenic variants [14].

In the present study, we investigated a family with two affected children who were suspected of having LGMD, but further pathologic and molecular studies using autozygosity mapping and NGS provide us the final diagnosis of a rare form of myopathy, Myopathy with extrapyramidal signs. To our knowledge it is the first report of this disorder in Iran and it also showed a novel mutation in causative gene, *MICU1.*

## Case presentation

**Fig. 1:**
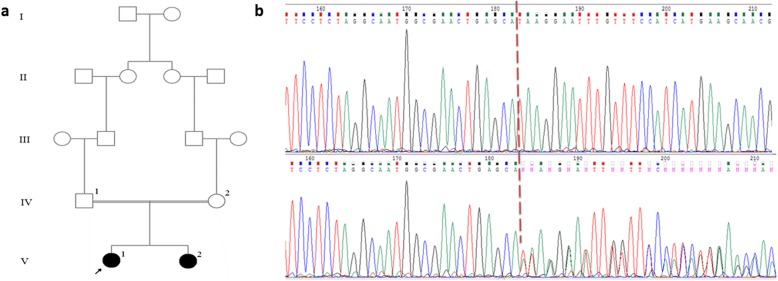
**a** Pedigree of the family with two affected children (filled symbols). **b** Sequence chromatograms of c.1294_1295delA mutation in the affected proband V1 are shown in homozygous form (up) compared to heterozygous form (her mother (IV2) (down)). Site of the deletion is shown with arrows

The proband was a 5-year-old girl who was referred to Kawsar Human Genetics Research Center (KHGRC) (Fig. 1). The affected proband had an affected sister and they were born to second cousin parents. This research has been performed in accordance with the Declaration of Helsinki; Informed consent was obtained from all family members and the study was approved by the ethical committee of the Pasteur Institute of Iran (No: 91/0201/10425). The proband was incidentally diagnosed with raised CK (5175 U/L) prior to muscle biopsy at the age of 5 but she was symptom free. Calf hypertrophy was also observed in the proband but she was ambulant at the age of 5 without positive Gowers sign. She showed high levels of CK up to 5175 U/L (normal < 145), LDH to 843 U/L and also increased level of ALT (105 U/L) and AST (71 U/L). She also had poor weight gain. Heart echocardiography showed a mild right side enlargement and mild pericardial infusion. EMG/NCV (electromyography and nerve condition velocity) study revealed short duration MUAPs in two upper and lower extremities tested muscles which was in favor of myopathic changes. The proband was followed up and examined at the age of 10 and she showed some extrapyramidal signs. The proband’s sister was 2 years old who was normal in her physical examination but showed elevated levels of CK (3442 U/L), AST (136 U/L), ALT (73 U/L), and LDH (799 U/L). Her EMG/NCV result was also normal. Both of them, the proband and her sister, had speech delay.

According to their clinical examination and family history, the affected siblings were suspected to have LGMD. Therefore, they were candidate of performing muscle biopsy studies.

**Fig. 2:**
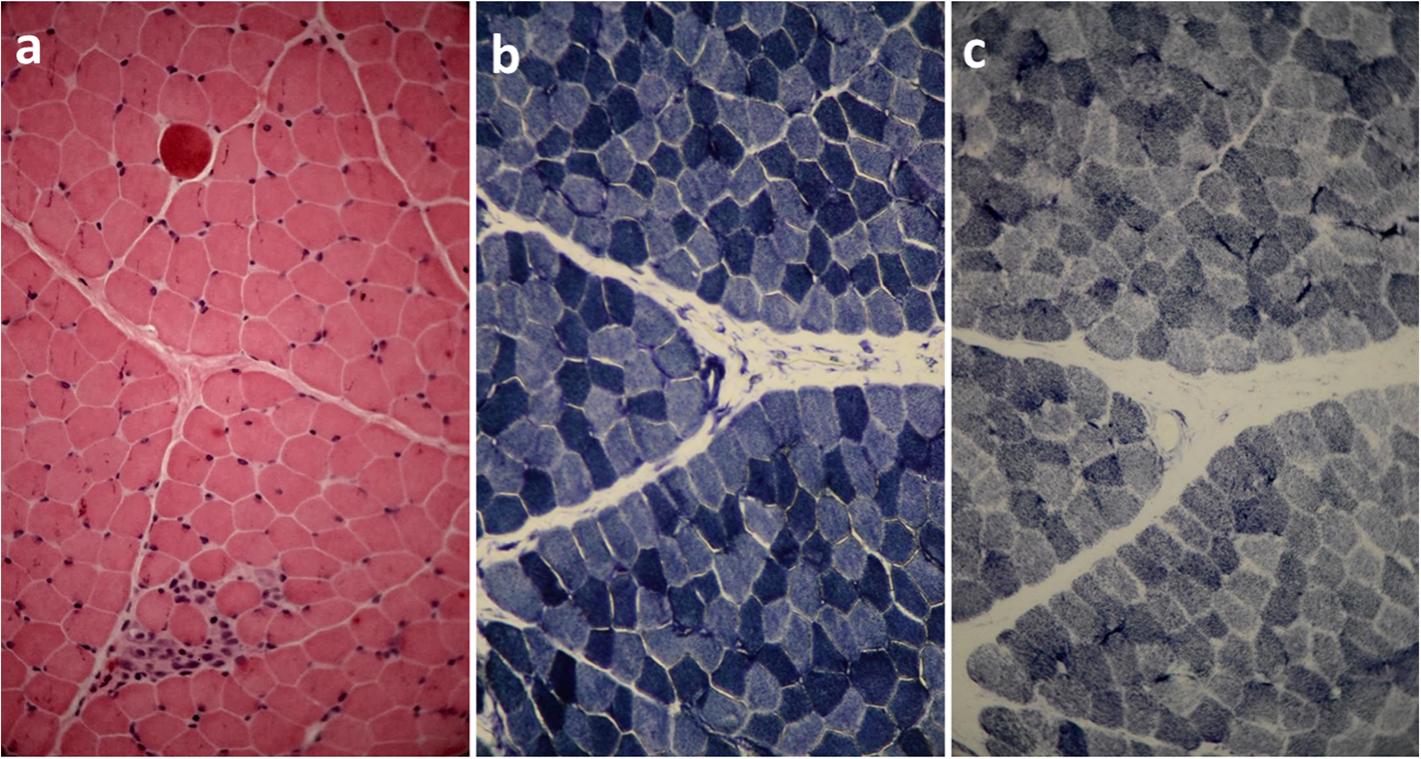
Muscle biopsy studies of the patient V1 when sataining with H & E (**a**), NADH-TR (**b**), SDH (**c**). All figures are (× 400) magnification

**Fig. 3:**
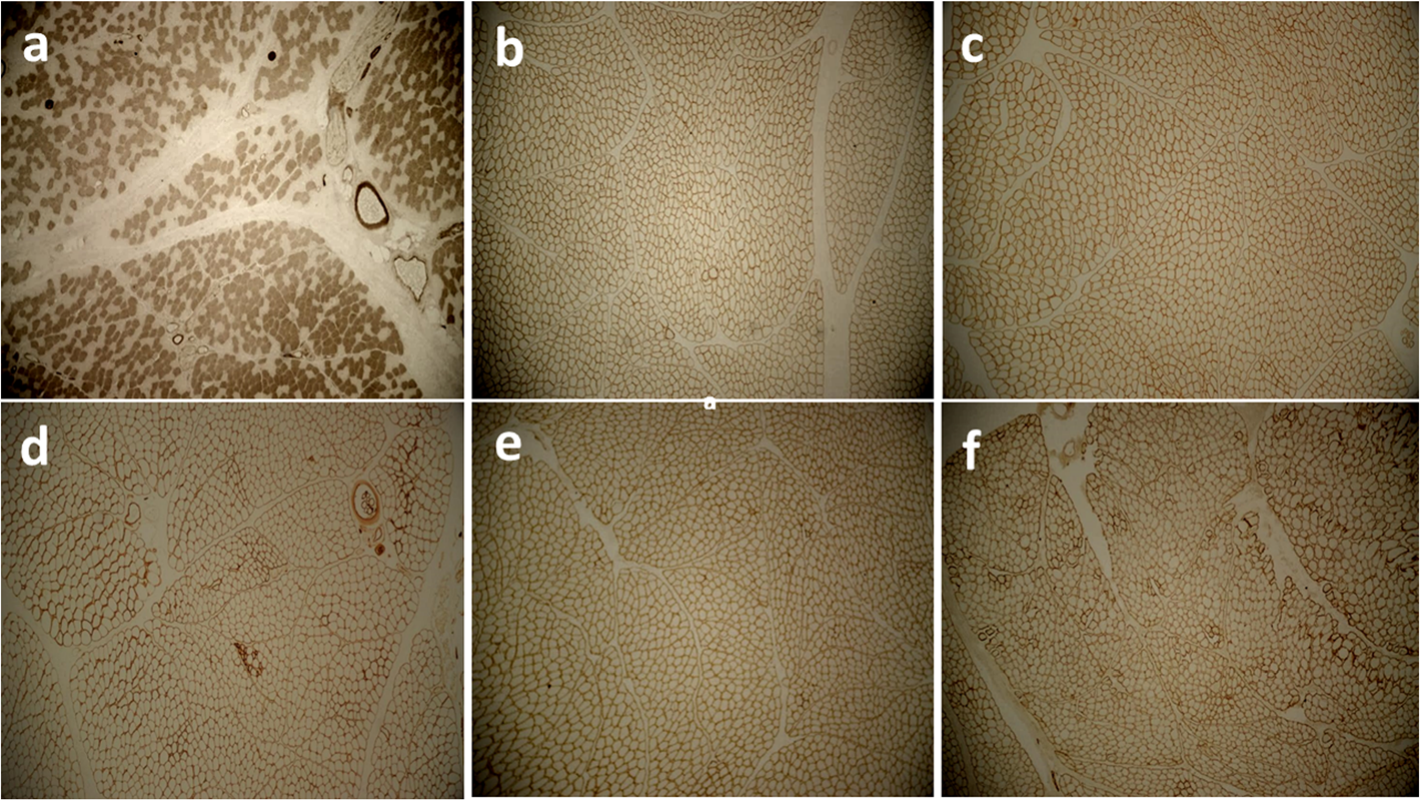
ATPase (the PH in this figure is 4.6) (**a**), DYS1 (**b**), DYS2 (**c**), Merosin (**d**), Alpha sarcoglycan (**e**), and Gamma sarcoglycan (**f**). All figures are (× 400) magnification

Muscle biopsy was performed on the proband at the age of 5. Fresh muscle specimen was taken from left deltoid of proband and quickly frozen in isopentane cooled by liquid nitrogen. Standard panel of special histochemical stains was used for sample analyzing [15]. Immunohistochemical staining was done using monoclonal antibodies against dystrophin (1, 2, 3), sarcoglycans (α, γ), dysferlin and merosin (Newcastle, UK). Beta-spectrin was also applied as a positive control. All antibodies were purchased from Novocastra Laboratories (Newcastle, UK). Microscopic study of hematoxylin and eosin (H&E) stains of tissue sections revealed slight variation in fiber size and dispersed, round and angular atrophic fibers with some small groups of necrotic or degenerative/regenerative fibers with rare myophagocytosis. Muscle fibers showed internalized nuclei but not prominently increased and hypercontracted myofibers were also seen (Fig. 2a). NADH-TR reaction revealed good differentiation of muscle fibers. Overall intermyofibrillar network seems regular (Fig. 2b). SDH reaction showed no prominent abnormal mitochondrial proliferation. Gomori-trichrome stains revealed no ragged-red-fiber. No red or rimmed vacuole have been observed. (Fig. 2c). ATPase histochemical reactions (at a PH of 9.4, 4.63, and 4.35) revealed type 1 fibers predominance. Most atrophic angular fibers were type 2, and no fiber type grouping was observed (Fig. 3a). Immunohistochemistry (IHC) staining of sarcolemmal proteins with monoclonal antibodies against DYS (dystrophin) 1, 2, and 3, sarcoglycans (α and γ), dysferlin, merosin and beta spectrin showed that almost all fibers were labeled which is suggestive of mild myopathic atrophy with few dispersed or small groups of degenerative/regenerative fibers but no endomysial fibrosis, no adipose tissue replacement, and no inflammation (Fig. 3b-f).

Ruling out the most common forms of LGMD, haplotype analysis has been done. Salting out procedure was used to extract genomic DNA from the peripheral blood samples of all the family members [16]. Haplotype analysis was performed using four STR markers linked to six genes of naming *CAPN3, DYSF, SGCA, SGCB, SGCG,* and *SGCD* in two multiplex PCR sets for all family members but no linkage was observed. Mutations in the mentioned genes are responsible for the most prevalent form of limb-girdle muscular dystrophies. DNA sequencing, interpretation and fragment analysis were done using methods described previously [17].

The blood sample of the proband was sent to Centogene (Rostock, Germany) for whole exome sequencing (WES) analysis. Enrichment was carried out using Nextera Rapid Capture Exome kit following the manufacturer’s protocol (Nextera Rapid Capture Exome, Illumina) and WES was performed by the Illumina HiSeq 2500 system sequencing platform (Illumina, San Diego, CA, USA) for DNA sequencing of the generated enriched library to an average coverage depth 70-100X. NGS results showed a novel homozygous deletion c.1295delA in exon 13 of the *MICU1* gene (NM_006077.3) [1]. This variation leads to a frameshift and cause premature stop codon in 8 following amino acids. In order to interpret pathogenicity of variants and predicting their effect on the protein structure, different software were used such as CADD (Combined Annotation Dependent Depletion) [18] and Mutation Taster [19]. According to the mutation taster, it is a disease causing mutation and its CADD score is 35.

Confirming the results of the WES, the Sanger sequencing was done in the proband and all her family members. As it was expected, the variation was in homozygous in two affected individuals and parents were carrier for this variation (Fig. 1b). These results indicated the co-segregation of the *MICU1* gene variant with the disease phenotype in this family. The timeline for diagnosis of the proband in revealed in Table 1.

**Table 1:** Timeline of diagnosis the proband

2012/06/25	Age at onset
2012/09/26	Physical examinations
2012/10/30	CK test and EMG/NCV
2012/12/05	Heart echocardiography
2013/07/27	Muscle biopsy study
2014/09/20	The first reffering for genetic diagnosis
2014/10/20	Autozygosity mapping
2016–2017	NGS analysis and Sanger sequencing
2017/03/05	Final Diagnoses

## Discussion and conclusions

There is little information available about the patients affected by myopathy with extrapyramidal signs worldwide, which is due to its low prevalence and low clinical specificity which cause overlapping clinical and pathological features with other motor disorders [8], therefore there are few reports available about this disorder. Logan and his group studied 11 patients with MICU1 pathogenic variants from five Pakistani families and 4 patients from two Dutch families in 2014 [1]. In the other study, 2 MPXPS patients with identified mutations were reported from the United Kingdom [8]. According to a study from Qatar, 13 patients from 10 consanguineous Middle Eastern Arab families showed recessive mutations in the *MICU1* [9].

Since consanguineous marriage is common in Iran, autosomal recessive forms of LGMD is more prevalent, therefore we ruled out the most common types of LGMDR by IHC study and autozygosity mapping. Thereupon we used NGS and found a novel deletion mutation (c.1294-1295delA) in the exon 13 of the *MICU1* gene. This homozygous deletion produced a premature stop codon which results in decreasing the length of the protein from 478 to 438 amino acids (p.Asn432Ilefs*8). The mutation located in the canonical EF4 calcium binding motif of MICU1 protein. This motif is located in the c-lobe presented in the intermembrane space (IMS) of mitochondria and contains one helix-loop-helix protein structure that has the ability to bind to one Ca^2+^ ion, Therefore our identified mutation may disrupt mitochondrial Ca^2+^ uptake [20–22]. Since amino acid Cys 465 has a role in heterodimer formation with MICU2 through a disulfide bond, removing this region by the mutation observed in our patient can abolish this function [23]. According to the joint consensus recommendation of the ACMG for the interpretation of sequence variants [24], this variant is a pathogenic one because of following evidences: 1. The nonsense and frameshift mutations are in a null variants category where they were predicted as a very strong evidence of pathogenicity (PVS1), 2. Absence the variant from control in population databases such as Exome Sequencing Project, 1000 Genomes Project, or Exome Aggregation Consortium is a moderate evidence for pathogenicity (PM2), 3. In-frame deletion in none repeat areas resulting changes in the protein length is considered a moderate proof for pathogenicity (PM4), 4. Several or various lines of computational data interpreted a deleterious effect on the gene (PP3), 5. The patient’s phenotypes are highly close to MPXPS (PP4).

Few mutations have been reported to lead the disruption of *MICU1* gene [25]. In a study by Logan et al. two loss of function mutations, c.1078–1G > C and c.7411G > A, located outside of the calcium binding motif of MICU1 reported in a cohort of 15 patients from 10 families with the disease phenotype [1]. Another study by Lewis-Smith et al. showed a homozygous deletion of exon 1 of *MICU1* in two cousins with no identifiable level of MICU1 protein in fibroblasts [8]. According to the latest report of the disease by Musa et.al a nonsense variant, c.553C > T (p.Q185*), was reported as a homozygous status in 12 patients and compound heterozygous in 1 patient that located in the N-terminal domain of *MICU1* gene [9].

To our knowledge, it is the first report of a myopathic patient with extrapyramidal signs from Iran and this patient has a novel mutation. Developing methods like autozygosity mapping and NGS which needs only the blood sample can overcome the limitations of invasive techniques such as immunohistochemistry which needs muscle biopsy sample. Since NGS is a high-throughput method, it can investigate all exons of the patient and it can conquer the limitation of autozygosity mapping which is restricted to some special genes. But it is obvious, using these techniques all together can confirm our diagnosis, specially application of haplotype analysis in prenatal diagnosis as an indirect method of diagnosis can corroborate our results.

## Supplementary information

**Additional file 1.** Sequencing data.

## Data Availability

All data generated or analyzed during this study are included in this published article and its supplementary information files.

## Abbreviations

MPXPS: Myopathy with extrapyramidal signs
NGS: Next Generation Sequencing
LGMD: Limb-girdle muscular dystrophy
STR: Short tandem repeats
WES: Whole exome sequencing
MICU1: Mitochondrial calcium uptake 1
ACMG: American College of Medical Genetics and Genomics
KHGRC: Kawsar Human Genetics Research Center
CADD: Combined Annotation Dependent Depletion
CK: Creatine Kinase
LDH: Lactate Dehydrogenase
EMG/NCV: Electromyography and nerve condition velocity
IHC: Immunohistochemistry
DYS: Dystrophin
IMS: Intermembrane space
