# Supplementary material for: Reporting a rare form of myopathy, myopathy with extrapyramidal signs, in an Iranian family using next generation sequencing: a case report
Source: BMC Med Genet. 2020 Apr 15;21:77. doi: 10.1186/s12881-020-01016-y (PMC7158096; doi:10.1186/s12881-020-01016-y)
Supplement: Supplementary file 1 — Additional file 1. Sequencing data. [file 12881_2020_1016_MOESM1_ESM.docx]

**Sequencing data:**

**Affected child:**

NNNNNNNNNNNANGANANCTAATGAATTCTAGGANAAGAGAATTGAGTTTGGCTGTGGAGAATGTCTGGGCTTTGAAATCCACTTTGTGTGGACAATGGAATTATATCAGTGGTGTTGTTCCAAGGCCAACACTTATCCCTGTTGTTTTTGCCTCTTCCTCTAGGCAATGGCGAACTGAGCATAAGGAATTTGTTTCCATCATGAAGCAACGGCTGATGAGAGGCCTGGAAAAGCCCAAAGACATGGGTTTCACTCGCCTCATGCAGGCCATGTGGAAATGTGCACAGGAAACTGCCTGGGACTTCGCTTTACCCAAACAGTAACCCCACACTGCAAGAGGGGACCCCTCCACCCCCAGTACCCTGGACCCCCTCCGCAGAGTCTCGGCAGAGCCCTTTGTGCTGCTGCTTCTGGAAGTAGTCTCCCTTCCTCCCGGGATGACCTCNGNAN

**Affected child's mother:**

NNNNNNNNNNNNNGANAAACTAATGAATTCTAGGANAAGAGAATTGAGTTTGGCTGTGGAGAATGTCTGGGCTTTGAAATCCACTTTGTGTGGACAATGGAATTATATCAGTGGTGTTGTTCCAAGGCCAACACTTATCCCTGTTGTTTTTGCCTCTTCCTCTAGGCAATGGCGAACTGAGCANNANGNANTTNNTTNCNNNNNNNANNNANNGNNNNANNNANGNCNGGNAAAANCCNAANAANNGGGNTTCCNTCCNCCCNNGGNNGNCNNGGNGNAANGGNNNNNNGNAANNNGCNNGGAANTNNNNTTNNCCNAANNNNWANCCCNNNNNNNNANAAGGGNNCCCNNCNNCCCCNNNNNCCNNGNNCCCCNNCNNNNAANNNNNGNNNAANCCNTTNNNSNNNNNNNTNNNGNARNNNNNNNCCNTNCNNCCNGGNNNNNCNNNNNNNNYNNNNNN

**Affected child's father:**

NNNNNNNNNNNANGANANCTAATGAATTCTAGGANAAGAGAATTGAGTTTGGCTGTGGAGAATGTCTGGGCTTTGAAATCCACTTTGTGTGGACAATGGAATTATATCAGTGGTGTTGTTCCAAGGCCAACACTTATCCCTGTTGTTTTTGCCTCTTCCTCTAGGCAATGGCGAACTGAGCANWANGNANTTNNTTNCNNNNNNNANNNANNGNNGNNNNNANGNCNNGNAAANNCCNAANAANNGGGNTTNNNTCCNCNNNNNNNNGNCNNGGNGNANNNNNNNNNNGNAANNNGCNNGNNANTNNNNTTNNCCNAANNNNNANCCCNNNNNNNNANAAGGNANCCCNNCNNCCCCNNNNNCCNNGNNCCCCNNCNNNNNANNNNNGNNNAANCCNTTNNKNNNNNNNNTNNNGNANNNNNNNNCCNTNCNNCCNGGNNNNNNNNNNNNNNNNNNNNAN
